# A situation analysis of child delivery facilities at primary health centers (PHCs) in rural India and its association with likelihood of selecting PHC for child delivery

**DOI:** 10.1186/s12913-021-07254-x

**Published:** 2021-11-08

**Authors:** Akif Mustafa, Chander Shekhar, Neha Shri

**Affiliations:** 1 International Institute for Population Sciences (IIPS), Mumbai, 400088 India; 2 Department of Fertility Studies, International Institute for Population Sciences (IIPS), Mumbai, India

**Keywords:** Primary health center, PHC, Place of delivery, India, NFHS

## Abstract

**Background:**

Primary Health Centers (PHCs) are crucial in providing primary and secondary level healthcare services in rural India. Despite immense efforts and huge funding, a very small proportion of deliveries are carried out at PHCs. The present study aims to explore the availability of facilities at PHCs and its association with likelihood of delivering the child at PHC.

**Methods:**

We extracted PHC level health infrastructure data from Health Management and Information system (HMIS) and created ‘Facility Index’ using exploratory factor analysis. We merged the ‘Facility Index’ with data of the 4th National Family Health Survey (NFHS-4) to explore the relationship between availability of facilities and healthcare-seeking behavior. Bivariate analysis and multilevel logistic regressions were employed to analyze the association between Facility Index and the likelihood of delivering the child at PHC.

**Results:**

Availability of facilities (Facility Index) was found to be positively associated with utilization of PHC for childbirth but up to only a certain level of Facility Index. Women living in districts with ‘good’ Facility index were having 2.45 (OR = 2.45; 95% CI: 2.12–2.84) times higher odds of delivering the child at PHC compared to women living in districts with ‘very poor’ Facility Index; however, the odds ratio decreased to 2.11 (95% CI: 1.83–2.43) for ‘Very Good’ Facility Index. The regression line and predicted probabilities also exhibited similar results.

**Conclusion:**

Based on the findings, we conclude that improvement in availability and quality of facilities might help in improving healthcare utilization from PHCs up to a certain level.

## Introduction

The adoption of Sustainable Development Goals (SDGs) has reaffirmed the reduction of preventable maternal and newborn deaths as global health priorities. With the Alma-Ata declaration on primary health care and Bhore Committee proposal (1946), health care services in India have been more effective and equitabl e[1]. In past few decades India has made substantial progress in the reduction of maternal and neonatal mortality rate. In a developing country like India, there is a widespread and growing demand for Primary health care. The provision of Maternal and Child Health services is an integral part of the service package to be provided by the Primary Health Centers (PHC).

One of the key strategies to scale back maternal mortality ratio (MMR) is the promotion of institutional deliveries to make sure that women have safe parturition car e[2]. Public health facilities are the main healthcare provider in India, especially for rural population and for the people of low socio-economic status. Recognizing the importance of facility-based maternal and newborn care, Primary Health Centers (PHCs) were established, strengthened, and modernized in accordance with Indian public health standards (IPHS )[3]. In order to increase public healthcare utilization, provision of operationalizing 50% of the PHCs as 24-h functional units was made under Reproductive and Child Health – II (RCH-II) programme. PHCs are also responsible for providing round the clock delivery services, including normal and obstetric emergency care, neonatal care services, and referrals. As PHCs are the first referral point in rural and remote areas, they are vitally important for the production of maternal and child healthcare services thereby reducing maternal and child mortality. Although, India has made tremendous progress in terms of health infrastructure and service provision, it is still lagging behind in meeting the healthcare demand in rural area s[4].

Despite the availability of free of cost, primary health care, maternal, and newborn services are underutilized at public health facilities. The PHCs are the backbone of healthcare service production in rural India. The key determinants of choosing PHCs for birthing care are good infrastructure, a clean and familiar environment, trust in the service provider, and behavior of the staf f[5]. Institutional delivery attended by a skilled birth attendant has been found to be associated with lower rates of maternal morbidity and mortality than home deliverie s[6, 7]. Delivery in health facilities also plays a critical role in prevention of stillbirths and increment in newborn surviva l[8]. Researchers have consistently shown that high cost of delivery care and geographical access to health care facilities are the major constraints for healthcare service utilization, particularly among the rural poo r[9, 10]. Previous studies have shown that, round-the-clock intrapartum services are available only in 60% of the PHCs, and in spite of concerted efforts to increase accessibility, approximately 30% of the PHCs do not offer any childbirth service s[11]. Availability of 24/7 PHCs has additional advantages in addressing the under-utilization of primary levels of health care [12]. Data shows that only about 40.5% of the total PHCs are functional 24* 7[13].

Despite the investment of an enormous amount of efforts and money, the data of NFHS-4 shows that only 7% of the deliveries in rural areas were conducted at PHC s[14]. It shows that people bypass PHC to deliver their child at a higher level or private medical facility in spite of significantly higher financial and non-financial costs. This bypassing is a phenomenon in which an individual chooses a farther located facility for healthcare-seeking instead of facilities close to their residenc e[15]. For example, instead of delivering the child at PHC, women prefer tertiary level facilities like Community Health Center (CHC), District Hospital, or private hospital. Which ultimately leads to higher expenditure, loss of time, opportunities, and wages. We did not found any study which has tried to analyze the condition of child delivery facilities at PHCs and its association with the choice of delivering the child PHC. Thus the present study makes an effort to analyze the availability/quality of childbirth facilities at PHCs and its association with the likelihood of delivering the child at PHC in rural areas of major states of India.

## Methods

### Data

The data used in this study are from HMIS (Health Management Information System) and the fourth round of the National Family Health Survey (NFHS-4, 2015–16). HMIS is a Government to Government (G2G) web-based Monitoring Information System, installed by the Ministry of Health & Family Welfare (MoHFW), Government of India. HMIS serves to monitor the National Health Mission of India and provides important insights in policy formation, program management, and key interventions. In addition, HMIS provides data on a range of health-related indicators like Maternal health, Child Health, Family planning, health infrastructure, et c[16] This study utilizes the data related to child delivery facilities at Primary Health Centers (PHCs) in rural India from the HMIS database 2017–18.

National Family Health Surveys are the Indian version of Demographic and Health Surveys (DHS), conducted by the International Institute for Population Sciences (IIPS), Mumbai. The fourth iteration of NFHS was conducted in 2015–16 under the stewardship of the Ministry of Health and Family welfares (MoHFW), Government of India. The NFHS-4 covered all 29 states and seven union territories. The survey provides data on a range of demographic and health indicators at the national, state, and district level, along with anthropometric data. The survey utilizes a two-stage sampling design to collect information from a sample of 699,686 women aged 15–49 years old from 601,509 households. The detailed information about the sampling design, methodology, response rate of the survey can be accessed for the NFHS-4 national repor t[14].

### Sample size

The present study focuses only on the rural areas of the country. The analysis of the present study is based on the data of 488 districts of India’s 17 major states. All the states having a hilly landscape were not included in the analysis because the availability of private facilities in rural areas of these hilly regions is negligible, and women have no choice between private and public facilities, so they have to deliver the child at a public facility or home. Therefore, in these regions percentage of child delivery at PHC will be automatically high. Hence we excluded these hilly regions from the analysis to avoid bias. Union territories were also excluded due to inadequate number of comments in the districts. The final sample size was 1,71,519 women aged 15–49 who gave birth to a child five years before the survey.

### Outcome variable

‘Source of delivery of the last child’ was the dependent variable for the analysis of the present study. The variable had numerous responses like Home, PHC, Community Health Center (CHC), private facility, etc. So for the analyses purpose, the variable was converted into a binary variable as:

**Table:** 

1	:	PHC
0	**:**	Other

### Explanatory variable

The principal explanatory variable was ‘Facility Index’ which we calculated using the HMIS data. The methodology of construction of Facility index is described below:

In the first place, we extracted data on five indicators related to facilities of child delivery at PHC from the HMIS database at the district level. The list of extracted variables is displayed in Table 1. All five variables were continuous in nature, varying from 0 to 100. For example, let us suppose there are 60 PHCs in a particular district and out of those, 15 are not having labor room; it means that 25% of the PHCs in that district are not having labor room. So the value of variable-1 for that district will be 75 (as 75% of the PHCs in the district have labour room facilities). It is clear from the above example that the values of the variables represent the percentage of PHCs in a district with a particular facility.

**Table 1:** Variables selected for construction of Facility Index

Variable 1	Labour room available at PHC
Variable 2	24-h facility of delivery at PHC
Variable 3	Labor room functioning or not?
Variable 4	doctors/nurses available for delivery
Variable 5	Condition of labor room (poor/not poor)

We calculated Cronbach’s Alpha to evaluate the internal consistency between the variables. The value of Cronbach’s alpha was found to be 0.687 showing a significant level of internal consistency between the variables. After that, we performed Exploratory Factor Analysis using the principal component method. The Factor Analysis produced five factors, among which Factor-1 had the highest eigenvalue (2.31) and was explaining approximately 76% variance of the selected variables. We selected Factor-1 for the construction of the Facility Index. According to quintiles, we divided factor scores into five categories and named the newly created variable as ‘Facility Index’. The five categories of the ‘Facility Index’ are:

**Table:** 

Value	Label
1	Very Poor
2	Poor
3	Average
4	Good
5	Very Good

### Controlled variables

The association between ‘delivery of child at PHC’ and ‘Facility Index’ was controlled for the following variables: Wealth Index (“Poorest”, “Poorer”, “Middle”, “Richer”, “Richest”), Women’s education level (“No Education”, “Primary”, “Secondary”, “Higher”), Age of the woman, Age of the husband, parity of the woman, sex of the head of the household, Religion, Caste, Health Insurance coverage and Occupation. Wealth Index is provided itself in the NFHS dataset, which is calculated based on possession of common household items and facilities[14].

### Statistical analysis

To explore the association between background variables and ‘delivery of child at PHC’, We performed bivariate analysis using simple chi-square test. The NFHS data is hierarchical in nature, i.e. the observations are nested in clusters. Because of this nature, the data suffer from intra-cluster correlation, i.e. the observations are likely to be correlated within the clusters. To account for this hierarchical nature of the data and intra-cluster correlation, we used two-level random-slope hierarchical regression model to assess the relationship between the outcome and the principle exposure variable. The clusters were set as level-2 in the regression mode l[17]. The advantage of multilevel model over simple regression model is that it not only takes intra-cluster correlation into consideration but it will also control for unobserved cluster level characteristics like cluster size, development level of the cluster, transportation facility, distance from the health facility etc.

We employed two regression models to explore the association between outcome and the main exposure variable. The first model was a univariate model, i.e. only ‘Facility Index’ was entered as the explanatory variable; this model was used to calculate crude odds ratios. The second model was a multivariate model i.e. the association between outcome and primary exposure variable was controlled for the background variables. Predicted probabilities of delivering child at PHC were calculated using post-estimation of the Model-2. All the statistical analyses were performed on STATA-1 6[18].

## Results

The status of availability of child delivery facilities at PHCs can be seen in Table 2. Out of 24,354 PHCs, labor room was not functioning at 6590 PHCs. Only 14,720 PHCs out of 24,876 were having 24-h facility of delivery, and doctor/Nurse/staff was not available in 4602 PHCs out of 17,233 PHCs.

**Table 2:** Status of availability of facilities at PHCs in rural India, HMIS (2017)

	Yes	No	Total
Labor room available	17,764	6590	24,354
24-h of delivery facility	14,720	10,156	24,876
Labor room functioning or not?	13,615	4149	17,764
Doctors/staff available for delivery?	12,631	4602	17,233
Poor condition of labor room?	14,312	2703	17,015

*Note: Total Number of PHCs are varying because of missing values*

Analyses indicated that in the rural areas of the selected states, a very small proportion (8.49%) of the deliveries were carried out at PHC, 24.24% of the deliveries were delivered at home, and approximately 21% of the deliveries were performed in private facilities (Table 3).

**Table 3:** Distribution of place of delivery for the last child, NFHS-4, (*n* = 171,519)

Place of delivery	n	weighted %
Home	47,038	24.24
PHC	14,866	8.49
Other public sector facilities/NGO hospital	81,305	46.3
Private Facility	28,310	20.97

*Note: These statistics are only of the selected 17 states, not the whole India*

Table 4 shows the distribution of ‘delivery of the last child at PHC’ with respect to Facility Index and background characteristics. It is evident from the table that the percentage of delivery at PHC was higher in districts having better facility index compared to districts with low facility index. In districts with ‘Good’ facility index, 11.13% of the deliveries were carried out in PHCs; on the other hand, this percentage was only 6.53% in districts with ‘very poor’ Facility Index. Percentage of women delivering at PHC was lower among mothers with higher wealth index compared to mothers with lower wealth index. Delivery at PHC was lowest among Sikh; only 2.61% of the Sikh mothers delivered their last child at PHC.

**Table 4:** Distribution of ‘Place of Last childbirth/delivery’ by Facility Index and background characteristics, NFHS-4 (*n* = 171,519)

	Last child delivered at	
	PHC (%)	Other (%)
facility index***		
Very poor	6.53	93.47
Poor	8.69	91.31
Average	10.59	89.41
Good	11.13	88.87
Very Good	9.94	90.06
Mother’s Education***		
No Education	11.88	88.12
Primary	9.27	90.73
Secondary	7.41	92.59
Higher	5.13	94.87
Wealth Index***		
Poorest	9.39	90.61
Poorer	10.05	89.95
Middle	8.67	91.33
Richer	6.3	93.7
Richest	3.94	96.06
Religion**		
Hindu	9.21	87.92
Muslim	7.26	88.1
Christian	8.07	84.95
Sikh	2.61	98.19
Caste*		
General	7.31	92.69
Scheduled Caste	8.69	91.31
Scheduled Tribe	9.77	90.23
OBC	8.49	91.51
**Total**	8.49	91.51

Note: *** *p* < 0.01,, ⁎⁎ *p* < .05, ⁎ *p* < .1, ns: Not Significant; *P*-values are calculated using chi-square test

**Fig. 1:**
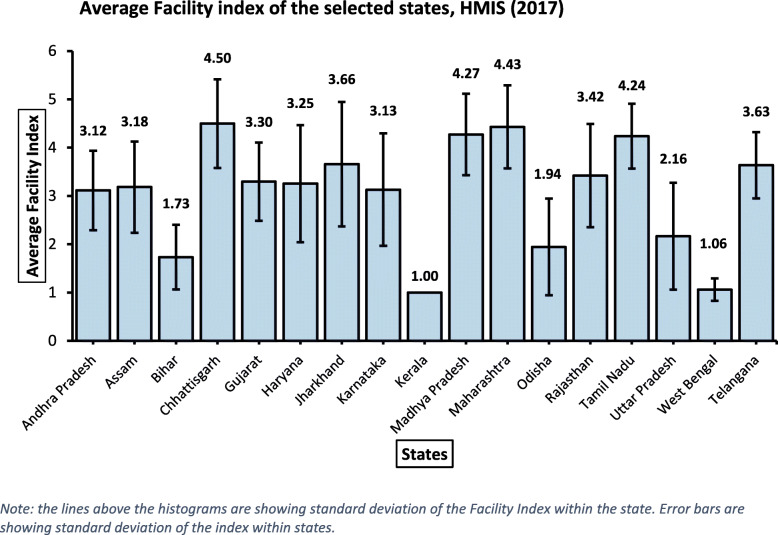
Average Facility index of the selected states, HMIS (2017). *Note: the lines above the histograms are showing standard deviation of the Facility Index within the state. Error bars are showing standard deviation of the index within states*

Figure 1 is showing state-wise histograms of the average Facility Index. From the graph, it can be visualized that the values of Facility Index were highly variable within states as well as between states. Some of the states like Maharashtra (4.43), Madhya Pradesh (4.27), Chhattisgarh (4.5), and Tamil Nadu (4.24) were having better facility scores compared to other states like Kerala (1.00), West Bengal (1.06), Bihar (1.73), Odisha (1.94), etc. In some of the states, the factor scores were consistent across the districts like Kerala (SD: 0), Maharashtra (SD: 0.86), and Tamil Nadu (SD: 0.67), on the other hand, the facility scores were highly inconsistent between the districts of some states like Jharkhand (SD: 1.29), Karnataka (SD: 1.16), Haryana (SD: 1.21) and Uttar Pradesh (SD: 1.11). One thing to be noted here is that having higher facility scores does not mean that the state/district has very good facilities/services of child delivery at PHC; rather it gives us a comparative insight that the states/districts with higher Facility Index have better delivery facilities at PHCs compared to states/districts with lower Facility Index.

**Fig. 2:**
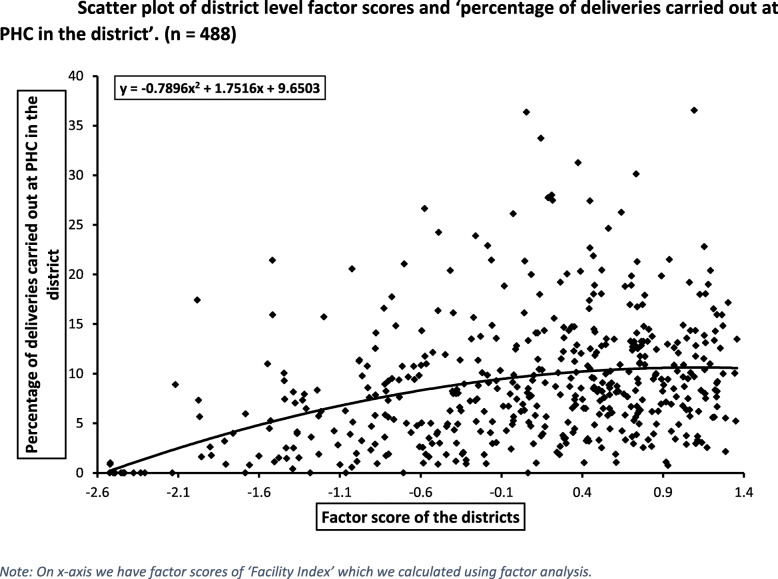
Scatter plot of district level factor scores and ‘percentage of deliveries carried out at PHC in the district’. (*n* = 488). *Note: On x-axis we have factor scores of ‘Facility Index’ which we calculated using factor analysis*

For graphical visualization, we created a scatter plot (Fig. 2) by plotting the ‘percentage of deliveries carried out at PHC in the district’ against the factor score of the districts which were calculated using exploratory factor analysis. From the graph, it can be visualized that initially, the percentage of deliveries carried out PHC increases with increase in the factor score, but after a certain level of factor score, there is no increase in the dependent variable. Thus, it gives us an indication that the quality and availability of delivery facilities at PHC is positively associated with choosing PHC as place of delivery, but after a certain level of quality/facility, the relationship between the two variables diminishes.

The results of the regression analysis have been summarized in Table 5. It can be seen from the table that the odds of delivering the child at PHC increases with the increase in score of the Facility Index. Women living in districts with ‘good’ Facility index were having 2.45 (OR = 2.45; 95% CI: 2.12–2.84) times higher odds of delivering the child at PHC compared to women living in districts with ‘very poor’ Facility Index. We can notice that the odds ratio is 2.45 for ‘Good’ facility index, but it decreases to 2.11 (95% CI: 1.83–2.43) for ‘Very Good’ Facility Index. Wealth Index was found to be having a negative association with the choice of delivering the child at PHC. Women with ‘Richest’ wealth Index had 67% (OR = 0.33; 95% CI:0.28–0.39) less odds of delivering the child at PHC compared to the women with ‘Poorest’ wealth index. The intra-cluster correlation was found to be 0.53, which indicates that 53% of the variation in choice of delivering the child at PHC was attributed to cluster/community level variables or the hierarchical nature of the data.

**Table 5:** Results of binary logistic regression assessing the Odds of last childbirth/delivery at PHC, NFHS-4, (*n* = 171,482)

	Model-1		Model-2	
	Odds Ratio	95% CI	Adj. Odds Ratio	95% CI
**Facility Index**				
Very Poor	1		1	
Poor	1.61***	1.39 - 1.85	1.53***	1.32 - 1.77
Average	2.35***	2.03 - 2.71	2.26***	1.96 - 2.62
Good	2.45***	2.12 - 2.83	2.45***	2.12 - 2.84
Very good	2.15***	1.87 - 2.48	2.11***	1.83 - 2.43
**Wealth Index**				
Poorest			1	
Poorer			1.06**	0.99 - 1.13
Middle			0.84***	0.78–0.91
Richer			0.56***	0.51 - 0.62
Richest			0.33***	0.28 - 0.39
**Religion of the head of the household**				
Hindu			1	
Muslim			0.82***	0.73 - 0.91
Christian			0.94 ns	0.75 - 1.19
Sikh			0.38***	0.61 - 0.16
**Caste of the head of the household**				
General			1	
SC			1.01 ns	0.92 - 1.11
ST			1.03 ns	0.93 - 1.16
OBC			0.98 ns	0.90 - 1.06
**Education (in number of years)**				
No Education			1	
Primary			0.91**	0.86 - 0.96
Secondary			0.86***	0.80 - 0.92
Higher			0.74***	0.67 - 0.81
Random effect	3.84		3.78	3.56 - 4.01
ICC	0.53		0.53	0.52 - 0.55

Note: *** p < 0.01,, ⁎⁎ p < .05, ⁎ p < .1, ns: Not Significant; ICC: Intra-cluster correlation

**Fig. 3:**
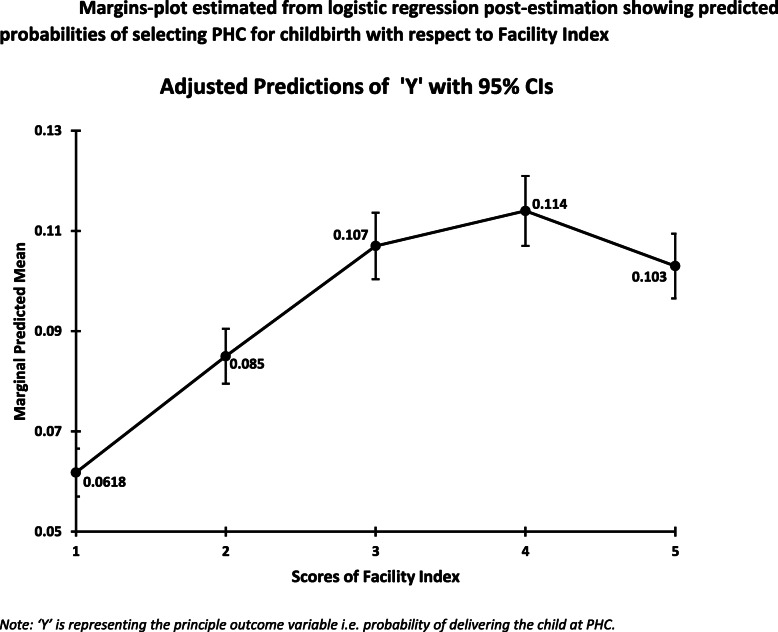
Margins-plot estimated from logistic regression post-estimation showing predicted probabilities of selecting PHC for childbirth with respect to Facility Index. *Note: ‘Y’ is representing the principle outcome variable* i.e. *probability of delivering the child at PHC*

Fig. 3 shows the margins plot of Facility Index categories calculated from the post-estimation of the multilevel logistic regression (Model-2). The graph illustrates that if all the facility index has the same Facility index of ‘1’ (very poor), then the probability of delivering the child at PHC is 6.1% (*p* = 0.0618; 95% CI: 0.067–0.057); On the other hand, if all the districts have Facility Index of ‘4’ (good) then the probability of delivering the child at PHC is approximately 11.4% (*p* = 0.114; 95% CI: 0.121–0.107). Again we can observe that the probability of delivering the child at PHC reduces to 10.3% (*p* = 0.103; 95% CI: 0.109–0.096) for the districts having a facility index of ‘5’ (Very Good).

## Discussion

PHCs were established to meet the demand for primary and secondary level healthcare services in rural areas of India. According to the estimates only 8.5% of the deliveries were carried out PHCs in rural areas of the selected states. There was a significant shortfall in childbirth-related facilities at PHCs. From statistical analyses, we found that the quality and availability of childbirth facilities at PHC is positively associated with people’s choice to deliver at PHC. Based on the findings of this study, it can be proposed that if the government takes necessary steps and improve the availability and quality of childbirth-related facilities at PHCs, the chances of choosing PHC as place of delivery are likely to increase. Apart from that, wealth index and education level were found to be having the strongest association with the outcome variable.

From the statistical analyses, we also observed that the likelihood of delivering the child at PHC does not increase after a certain level of delivery facilities at PHCs. It gives us an insight that the availability of facilities is not the lone factor associated with the choice of place for child delivery. Similar findings were found in a 2010 study, in which the authors claimed that economic status is the most important factor in deciding between private and public facilities for institutional deliver y[19]. Previous research shows that, social factors such as, behavior of staff, being attended without delay, religious barriers, and having access to social support during care, affect the choice of delivery. Due to data unavailability, we could not analyze the effect of accessibility/‘distance of PHC from the residence’ on the outcome variable. Accessibility to public healthcare facilities reflects the country’s progress in the health system and equity towards those who cannot afford it. In literature, we find that geographical access is a robust determinant of healthcare-seeking from public health facilit y[15, 20, 21]. However, in another study, the effect of geographical accessibility was found to be varying between the states: among four states of India (Andhra Pradesh, Gujarat, Bihar, and Rajasthan), accessibility to healthcare facility was found to be statistically significant only in Rajastha n[22]. Apart from the distance of PHC from the village, other variables like quality of roads, availability of transportation facility, ambulance facility, etc., are also important for easy accessibility and minimized delay. There is scope of a future study that can comprehensively analyze the effect of above-mentioned factors on choice of delivery.

In rural areas, having a 24-h delivery facility at PHCs has immense importance. Majority of the time, community health centers/tertiary care hospitals/private hospitals are situated far from the village due to which access to these facilities becomes very difficult in the night time. As a result, many prefer to give birth at home. Therefore, if PHCs provide 24-h delivery services, this will result in an increase in institutional deliveries, which will ultimately help to reduce maternal and infant mortality in rural areas of the country.

In rural India, perception plays a very important role in the utilization of service from any source. Negative perception about public facilities is likely to impact the choice of place of delivery. People of rural areas tend to negate the utilization of government facilities if they have the option to go for private health care even when the public facility is providing good quality of service because the public sector is often perceived as of low quality. There is another type of mentality prevalent among the rural area people with lower education levels is that they do not value institutional deliveries as much as health institutions do. People tend to believe that childbirth is a natural process and does not require medical intervention. The data of NFHS-4 shows that 39% of the women who delivered their last child at home believe that institutional deliveries are not necessary. According to a study, this belief is more common in north India, and we also observe that percentage of home deliveries is comparatively higher in north Indi a[14, 23]. These evidence show that in addition to quality care and high accessibility the rural population of the country needs to be educated and the government should enact policies to promote health education and increase awareness regarding institutional delivery.

Neelanjana Pandey, in her Ph.D. work ‘Access to public health facilities: A focus on maternal health care in rural Uttar Pradesh’, mentioned three dimensions of PHC accessibility: locational access, health personnel access, delivery facility, and infrastructure at PHC. In her study, she found that good quality and high accessibility were not associated with increased utilization of PHC for institutional deliveries. However, the study focused only on a state (Uttar Pradesh) of India, and the results of the study cannot be generalized for the whole countr y[24].

Out of pocket expenditure for healthcare seeking in the private sector is significantly high in the country. According to NFHS-4, in rural areas of the country, the average cost of delivery was 2947 rupees in the public facilities; on the other hand, in private facilities, this cost was 15,036 rupees which is approximately five times the cost in public facilit y[14]. Apart from that, under Janani Suraksha Yojana (JSY), the government of India provides monetary incentives to those women who deliver their child at public facilit y[25]. Still, we see that around 46% of the deliveries were either carried out at a private facility or at home, and only 7 % were carried out at PH C[14]. This poor utilization of PHCs for child delivery raises various concerns regarding the efficiency of PHCs in providing maternal healthcare in rural India.

### Limitations

Due to data limitations, we could not control the effect of various important factors on the association between outcome and exposure variable. For example, distance of PHC from the village, quality of roads, facility of ambulance at PHC, distance of higher-level medical facility, etc. these factors influence the people’s choice of place for healthcare seeking, but we could not include these variables in the analysis due to data unavailability. Another limitation was that for analysis of the present study we have utilized 2017 data of HMIS and merged it with NFHS (2015–16) data i.e., both the data set are of different time frame (2 years’ gap) assuming that there have not been any significant ground-level changes in healthcare utilization pattern between 2015 to 2017. These were the only limitations of the present study.

## Conclusion

From the results of this study and preceding discussion, it is clear that choice of delivery-place is influenced by numerous social, economic, and infrastructural factors. According to the findings of this study, improvement in availability of facilities is likely to increase the likelihood of delivering the child at PHC but up to only a certain extent. Thus, simply improving the availability of facilities alone will not significantly solve the problem of low utilization of PHCs for child delivery. To improve the use of maternal health services, it is critical to understand the social interaction at the community level and its influence on people’s attitudes and opinions regarding care-seeking. It is critical for Policymakers to understand the bypassing behavior of individuals and to analyze why people choose a specific facility for delivery when other feasible options are available.

## Data Availability

The data utilized for the present study is freely available in public domain through: https://www.dhsprogram.com/data/dataset/India_Standard-DHS_2015.cfm?flag=0

## Abbreviations

PHC: Primary Health Center
CHC: Community Health Center
EFA: Exploratory Factor Analysis
HMIS: Health Management Information System
IPHS: Indian Public Health Standards
NFHS: National Family Health Survey
RHS: Rural Health Statistics
NRHM: National Rural Health Mission
